# Contribution of Enteroviruses to Acute Central Nervous System or Systemic Infections in Northern Italy (2015-2017): Is It Time to Establish a National Laboratory-Based Surveillance System?

**DOI:** 10.1155/2020/9393264

**Published:** 2020-07-01

**Authors:** Antonio Piralla, Laura Pellegrinelli, Federica Giardina, Cristina Galli, Sandro Binda, Elena Pariani, Fausto Baldanti

**Affiliations:** ^1^Molecular Virology Unit, Microbiology and Virology Department, Fondazione IRCCS Policlinico San Matteo, Pavia, Italy; ^2^Department of Biomedical Sciences for Health, University of Milan, Milan, Italy; ^3^Department of Clinical, Surgical, Diagnostic and Pediatric Sciences, University of Pavia, Pavia, Italy

## Abstract

**Background:**

Enteroviruses (EVs) can cause infections and outbreaks of mild to severe diseases, such as central nervous system (CNS) and systemic infections. The contribution of EVs to acute CNS/systemic infections requiring hospitalization was assessed by analysing data extracted from virology laboratory database.

**Methods:**

Real-life data obtained from two molecular virology laboratories located in Northern Italy were retrieved from databases and analysed retrospectively. The queries used to extract the data were (i) requests for EV-RNA detection in clear cerebrospinal fluid (CSF) specimens collected from hospitalized patients with suspected acute CNS (including aseptic meningitis, encephalitis, and acute flaccid myelitis/paralysis) or systemic infections (sepsis-like illness or fever (≥ 38°C) of unknown origin), (ii) CSF samples collected from January 1st, 2015, to December 31st, 2017.

**Results:**

582 requests of EV-RNA detection in CSF samples collected from as many patients of any age were recorded. EV-RNA was detected in 4.5% of the CSF samples; 92.3% of EV-positive cases were patients < 15 years, 58.3% of whom were < 3 months. EVs circulated all-year-round, and the highest EV-positive rates were observed from May to August. The risk of EV infection and the relative illness ratio value among children < 1 − year − old were significantly higher than those observed for older patients.

**Conclusions:**

EV surveillance should be carried out for all pediatric patients < 15 years and especially children less than 1 year of age with clinically suspected CNS infection/systemic infections. The implementation of a laboratory-based surveillance established for analysing the virological data provided by laboratories that routinely perform EV molecular testing may enable us to determine the impact of EVs that can cause infections requiring hospitalization.

## 1. Introduction

Human Enteroviruses (EVs) belong to the *Enterovirus* genus of the *Picornaviridae* family and are widespread viruses transmitted through faecal-oral and respiratory routes or through contact with contaminated fluids and surfaces (1). Nowadays, more than 100 types of EVs have been identified which can have significant effects on human health (1); the clinical manifestations of EV infections may range from mild nonspecific conditions to severe diseases whose clinical characteristics are generally associated with the type of EV involved (2). EVs can cause severe central nervous system (CNS) infections, including aseptic meningitis, encephalitis, and acute flaccid myelitis/paralysis, especially among children under 15 years of age (1). Systemic infections (such as sepsis-like illness and fever of unknown origin) have also been reported among patients with EV infections (1). In general, the most severe outcomes and life-threatening complications of EV infections have been reported in children under 5 years of age (1, 2).

There is currently no vaccine or specific antiviral therapy against EVs (except against poliovirus and EV-A71). Nonpolio EV infections are not included in the list of notifiable diseases in Italy, and a nonpolio EV surveillance system has not yet been established; therefore, the impact caused by EVs and their epidemiological characteristics remain poorly defined. In the absence of a systematic nonpolio EV surveillance system, laboratory-based surveillance conducted by analysing the data provided by virological laboratories that routinely perform EV testing may enable us to determine the impact of EVs that can cause infections requiring hospitalization and promptly detect EV outbreaks (3).

In this retrospective analysis, the frequency and contribution of EVs to acute CNS/systemic infections requiring hospitalization were evaluated by analysing data extracted from the databases of two virology laboratories located in Northern Italy during 3 consecutive years.

## 2. Materials and Methods

### 2.1. Study Design

Data from real-life diagnostic activities of the Molecular Virology Unit, Fondazione IRCCS Policlinico San Matteo, Pavia, and the Department of Biomedical Sciences for Health, University of Milan, Milan, (Lombardy region, Northern Italy) were retrieved from diagnostic databases and analysed retrospectively.

The queries used to extract the data from the databases were (a) requests for EV-RNA detection, in clear cerebrospinal fluid (CSF) specimens collected from hospitalized patients in the case of suspicion of acute CNS infection (including aseptic meningitis, encephalitis, and acute flaccid myelitis/paralysis), or sepsis-like illness or fever (≥38°C) of unknown origin and (b) requests submitted from January 1st, 2015, to December 31st, 2017.

For routine EV detection, after RNA extraction from the CSF samples using a commercial kit (QIAamp MinElute Virus Spin, Qiagen, GmbH, Hilden, Germany), the samples were screened for the presence of EV genome by an in-house one-step real-time RT-PCR assay on a StepOnePlus Real-Time PCR system (Thermo Fisher Scientific, Inc., Massachusetts, USA) as previously described (4, 5).

The study was performed according to the guidelines of the Institutional Review Board on the use of biological specimens for scientific purposes in keeping with Italian law (art. 13 D.Lgs 196/2003). The study was conducted in accordance with the Declaration of Helsinki 1975, rev. 2000. The data were handled anonymously.

### 2.2. Statistical Analysis

The statistical analysis was performed using Open Source Epidemiologic Statistics (OpenEpi) for Public Health software (6). Categorical variables were expressed as numbers and proportions and were compared using the Chi-squared test or Fisher's exact test based on binomial distribution as appropriate. Continuous variables were expressed with mean and standard deviation (SD) or median and lower and upper quartiles (Q1 and Q3) and compared using the unpaired *t*-test. A *p* value < 0.05 was considered significant (two-tailed test).

The risk of infection was expressed as the number of patients with a laboratory-confirmed EV infection out of the total number of patients with a specific characteristic. The odds ratio (OR) and exact confidence limits (95% CI) were calculated using the Mid-*p* exact test assuming a normal distribution. The relative illness ratio (RIR) was expressed as the ratio of the percentage of EV-positive cases in the considered age group out of the percentage of the population of the Lombardy region belonging to the same age group; data on the annual population composition in the Lombardy region were obtained from Istituto Nazionale di Statistica (ISTAT) (7).

## 3. Results and Discussion

### 3.1. Result

A total of 582 requests for EV-RNA detection in CSF samples collected from as many patients were recorded during the three-year study period and were included in the analysis. Overall, 51.9% (*n* = 302) of the cases were males (Table 1). The median age of the patients was 40.2 years (Q1-Q3: 5.9-69.7 years; range: 0-97 years). As shown in Tables 1, 33.5% (*n* = 195) of the CSF samples were obtained from children (0-15 years) and 66.5% (*n* = 387) were obtained from adults (>15 years). 40.5% (*n* = 79) of the pediatric patients were aged 0-3 months, 10.3% (*n* = 20) 4-12 months, 25.1% (*n* = 49) 1-6 years, and 24.1% (*n* = 47) 6-15 years. 56.6% (*n* = 219) of the adult patients were aged 16-65 years, and 43.4% (*n* = 168) were > 65 years. The requests were evenly distributed across the three-year study period: 32.5% (*n* = 189) in 2015, 38.1% (*n* = 222) in 2016, and 29.4% (*n* = 171) in 2017 (*p* > 0.05).

During the study period, 26 out of 582 (4.5%) CSF samples were EV-positive (Table 1). EV was detected more frequently in males than in females (61.5% vs. 38.5%, *p* = 0.03) (Table 1). The median age of EV-positive patients was lower than that of EV-negative patients (3.7 years vs. 37.6 years; *p* < 0.001). As shown in Table 1, 92.3% (24/26) of the EV-positive cases were children < 15 years: 58.3% (*n* = 14) of them were aged 0-3 months, 12.5% (*n* = 3) 4-12 months, 16.7% (*n* = 4) 1-6 years, and 12.5% (*n* = 3) 6-15 years. Only two EV-positive cases (7.7%) were detected in adult patients aged 16-65 years; no EV-positive cases were detected among patients > 65 years (Table 1). The prevalence of EV-positive cases by the year of study was 5.3% (10/189) in 2015, 3.1% (7/222) in 2016, and 5.2% (9/171) in 2017, with no statistical differences (*p* > 0.05) (Table 1).

**Figure 1:**
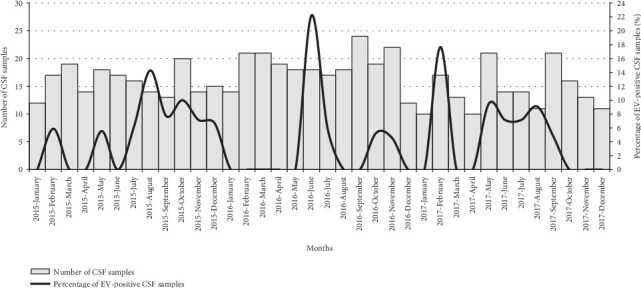
Temporal (monthly) distribution of EV-positive CSF samples (2015-2017).

A seasonal pattern of EV-positive cases was observed in the three-year study period (Figure 1). In fact, the highest EV-positivity rate was observed from May to August (15/26, 57.7% of all the EV-positive cases) each year. As shown in Figure 1, a statistically significant increase in the EV-positivity rate was observed in June-July 2016 (5/35; 28.1% vs. 21/547; 3.8%; *p* = 0.01) and in February 2017 (3/17; 17.6% vs. 23/565; 4.1%; *p* = 0.03), respectively.

The risk of EV infection among children under 6 years of age was 14-fold (OR: 14.1; 95% CI: 5.5-42.7) higher than the other patients. Children < 1 year had a risk of EV infection nearly 11-fold (OR: 10.8; 95% CI: 4.7-26.3) higher than among the older age groups. The RIR value was 3.8 (95% CI: 2.7-6-3) in children under 6 years and increased to 10.1 (95% CI: 17.8-31.4) when only children aged less than 1 year were considered. The risk of contracting an EV infection in early summer (May-August) was 2.5-fold (OR = 2.5; 95% CI: 1.2-5.5) higher than in other months.

#### 3.2. Discussion

In this retrospective study, we assessed the frequency and distribution of EV infections among patients hospitalized with acute CNS or systemic infections over three consecutive years (2015-2017). In our series, EVs were detected in 4.5% of cases of acute CNS/systemic infections, at frequencies similar to those observed in other studies. In fact, during a Scottish 5-year study (2005-2010) aimed at evaluating the molecular epidemiology of EVs among hospitalized patients, the prevalence of EV detection in CSF samples was 5.3% (8), while the average annual percentage of EV-positive specimens of all types was 6.5% in the Netherlands during the period 1991-2006 (9). It has been observed that children are at an increased risk of contracting EV infections (10). This finding is confirmed by our results: although approximately two-thirds of the CSF samples analysed were collected from adult patients, almost all (92.3%) of the EV-positive cases were identified in pediatric patients aged <15 years. The highest prevalence of EV-positive samples (58.3%) was identified in children under 3 months of age, which is a similar value to those reported in the UK and France where the highest proportion of all EV infections were detected in children < 3 months (8, 11). Similar to other studies (10, 11), in our series, EV was detected more frequently in males than in females (61.5% vs 38.5%, *p* = 0.03). As also observed in Europe and the US, throughout the study period, the frequency of EV infections peaked from May to August each year (10, 12), when the risk of EV infection was over twofold higher than in other months. A sudden increase in EV detection was observed in June-July 2016 and in February 2017, when the rates of detection of EV in the CSF specimens analysed reached 28% and 17.1%, respectively.

According to the US National Enterovirus Surveillance System, children under 1 year of age are at an increased risk of unfavorable outcomes of EV infections (11). It is important to note that in our series, both the risk of EV infection and the relative illness ratio value among children < 1 year were significantly higher than those observed in older patients, thus indicating that this age group is the most affected by EV infections.

Laboratory-based surveillance has been defined as one of the pillars of monitoring infectious diseases trends and relies on data produced in clinical and public health laboratories (13). As conceptualized in the framework of the “TYPENED” (14) and “ICARES” data-sharing system (3) in the Netherlands, we may be able to determine the viral distribution among hospitalized patients by analysing the virological data extracted from diagnostic databases of laboratories routinely performing EV molecular testing. The collection of a minimum dataset (including age, gender, hospitalization, type of sample collected for the analysis, and clinical symptoms) using a centralized real-time database system and software may enable us to identify spatial, temporal, and demographic changes in EV prevalence and to promptly identify any EV outbreak, as recently demonstrated in the Netherlands (3, 15).

The main limitation of this study is that it relies on data that were not purposely collected to describe EV molecular epidemiology. As it is a retrospective study, it has a number of intrinsic limitations: only data from patients with a specific query for EV-RNA detection in clear CSF specimens with clinically suspected EV infections were available and no other biological samples were systematically investigated. Moreover, in this retrospective study, the genotypes of detected EV could not be defined due to lack of residual samples. Lastly, the data analyzed in this study were extracted from diagnostic databases of virological laboratories that do not include results on biochemical parameters, neurological outcomes, or clinical follow-up.

## 4. Conclusions

According to our results, EV surveillance should be carried out for all pediatric patients < 15 years and especially children less than 1 year of age with clinically suspected CNS infection/systemic infections, particularly during the summer/autumn period when EVs are more likely to circulate among the population.

Performing routine EV molecular testing could accelerate diagnosis. Although as yet no specific vaccines or antivirals are currently available for EV infections, the administration of broad-spectrum antibiotics or steroids would be suspended in EV-positive patients, ameliorating the clinical treatment accuracy.

The establishment of national laboratory-based surveillance for analysing the virological data obtained from laboratories that routinely perform EV testing can help in defining the impact of EV on infections requiring hospitalization and may provide us with scientific evidence on the clinical impact and epidemiology of nonpolio EVs.

## Figures and Tables

**Table 1:** Distribution of CSF samples and EV-positive CSF samples by age group, gender, and year of study.

		No. (%) of CSF samples	No. (%) of EV-positive CSF samples
Median age [Q1-Q3] (year)		40.2 [5.9-69.7]	3.7 [0-4.5]
Gender	Male	302 (51.9%)	16 (61.5%)
	Female	280 (48.1%)	10 (38.5%)
Children	0-3 months	79 (13.6%)	14 (53.8%)
	4-12 months	20 (3.4%)	3 (11.5%)
	1-6 years	49 (8.4%)	4 (15.4%)
	6-15 years	47 (8.1%)	3 (11.4%)
	Total	195 (33.5%)	24 (92.3%)
Adults	16-65 years	219 (37.6%)	2 (7.7%)
	>65 years	168 (28.9%)	0 (0%)
	Total	387 (66.5%)	2 (7.7%)
Year of study	2015	183 (32.5%)	10 (38.5%)
	2016	222 (38.1%)	7 (26.9%)
	2017	171 (29.4%)	9 (34.6%)
	Total	582 (100%)	26 (100%)

## Data Availability

The data used to support the findings of this study are available from the corresponding author upon request.
